# Case Report: Eosinophilic Bronchiolitis With Eosinophil ETosis in Mucus Plugs Successfully Treated With Benralizumab

**DOI:** 10.3389/fphar.2021.826790

**Published:** 2022-01-13

**Authors:** Hisashi Sasaki, Jun Miyata, Akiko Irie, Ayako Kuwata, Yuji Kouzaki, Shigeharu Ueki, Akihiko Kawana

**Affiliations:** ^1^ Division of Infectious Diseases and Respiratory Medicine, Department of Internal Medicine, National Defense Medical College, Saitama, Japan; ^2^ Department of Internal Medicine, Japan Self-Defense Forces Central Hospital, Tokyo, Japan; ^3^ Department of General Internal Medicine and Clinical Laboratory Medicine, Akita University Graduate School of Medicine, Akita, Japan

**Keywords:** benralizumab, Charcot-Leyden crystal, eosinophil, eosinophilic bronchiolitis, mucus plug, ETosis

## Abstract

Eosinophilic bronchiolitis is a rare allergic disorder caused by eosinophilic inflammation in the bronchioles of the lungs. An effective treatment strategy is needed in cases resistant to steroids. However, its pathophysiology remains unclear owing to the limited number of cases. We herein present the case of a 31-year-old man who experienced eosinophilic bronchiolitis with eosinophil ETosis (EETosis) in the mucus plugs. The patient was diagnosed with asthma. His respiratory symptoms worsened with eosinophilia when treated with the standard asthma regimen, including inhaled corticosteroids, long-acting β2-agonist, long-acting muscarinic antagonist, and leukotriene receptor antagonist. Chest computed tomography revealed bronchial wall thickening and centrilobular nodules in the lower lobes of both lungs. Bronchoscopy showed obstruction of the subsegmental bronchus with mucus plugs. Histological analysis demonstrated abundant eosinophils in the mucus plugs. Cytolytic eosinophils together with Charcot–Leyden crystal formations and deposition of major basic proteins were also observed, indicating the occurrence of EETosis. Introduction of benralizumab, an anti-interleukin-5 receptor α antibody, successfully controlled the patient’s condition and reduced the amount of systemic corticosteroids administered. Our findings confirm that this antibody strongly decreases airway eosinophils in patients with severe asthma. Thus, benralizumab might be an optimal therapeutic agent for the treatment of mucus plug-forming and/or EETosis-occurring eosinophilic lung diseases, including eosinophilic bronchiolitis.

## Introduction

Eosinophilic bronchiolitis is a rare condition caused by eosinophilic inflammation of the bronchioles in the lungs. In a previous report, this syndrome was defined as follows: 1) a blood eosinophil count of >1,000/µl and/or a bronchoalveolar lavage (BAL) eosinophil differential cell count of >25%; 2) persistent airflow obstruction on lung function tests not modifiable after 4–6 weeks of inhaled corticosteroid therapy (2,000 μg/day beclometasone or equivalent); and 3) lung biopsy showing inflammatory bronchiolitis with prominent bronchiolar wall infiltration by eosinophils and/or characteristic direct high-resolution computed tomography (HRCT) features of bronchiolitis (poorly defined centrilobular nodules, branching opacities, and tree-in-bud pattern) (Poletti, 2013). However, the pathogenesis of this disease remains unclear because only a limited number of cases have been reported in literature. Systemic corticosteroids are often used, but some cases manifest steroid resistance (Poletti, 2013; Tang et al., 2015). We herein present a case of steroid-resistant eosinophilic bronchiolitis in a patient with asthma who was successfully treated with benralizumab, an anti-interleukin 5 (IL-5) receptor α (IL-5Rα) antibody.

## Case Description

A 31-year-old man with a non-smoking history was diagnosed with asthma along with paroxysmal cough, sputum, dyspnea, and wheezing. His asthma was well controlled with inhaled corticosteroid/long-acting β2-agonist (budesonide formoterol, 1,280 µg/36 µg/day), long-acting muscarinic antagonist (tiotropium, 2.5 µg/day), and leukotriene receptor antagonist (montelukast, 10 mg/day). Three years after the diagnosis of asthma, his cough, sputum, and dyspnea worsened, with a high blood eosinophil count of 1,222/μl. Administration of oral prednisolone (20 mg/day) for 1 week temporarily improved his respiratory symptoms. However, the symptoms recurred after cessation.

**FIGURE 1:**
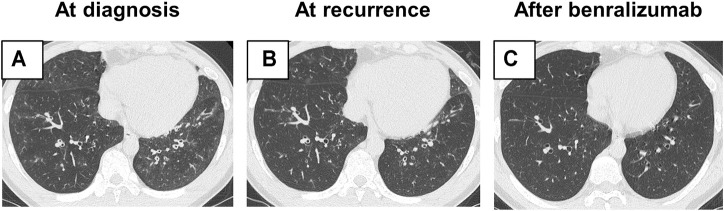
Radiological findings. **(A)** High-resolution computed tomography (HRCT) findings on admission showing bronchial wall thickening and centrilobular nodules in the lower lobe of both lungs. **(B)** At recurrence, HRCT showed radiological findings similar to those seen on admission. **(C)** After the addition of benralizumab, bronchial wall thickening and centrilobular nodules improved.

He was therefore hospitalized with these respiratory symptoms. On admission, he had fever (38.1°C) and hypoxia (SpO_2_: 93% at room air). Laboratory tests revealed elevated levels of blood eosinophil count (1,976/µl), total IgE (529 IU/ml), and fractional exhaled nitric oxide (FeNO; 165 ppb). Myeloperoxidase-anti-neutrophil cytoplasmic antibodies and fungus-specific IgE antibodies (*Aspergillus*, *Candida*, and *Alternaria*) were negative (Table 1). Pulmonary function test results showed mild obstructive ventilatory impairment with a percent forced expiratory volume in one second of 73%. Asthma Control Test (ACT) score is 15. Reverse transcription-polymerase chain reaction assay of the sputum specimen for the presence of severe acute respiratory syndrome coronavirus 2 showed a negative result. HRCT findings showed bronchial wall thickening and centrilobular nodules in the lower lobes of both lungs (Figure 1A).

**TABLE 1:** Laboratory findings on admission.

Peripheral blood		Biochemistry	
White blood cells	11,360/μl	Total bilirubin	0.63 mg/dL
Neutrophil	56.4%	Aspartate transaminase	29 IU/L
Lymphocyte	20.7%	Alanine transaminase	34 IU/L
Basophil	0.4%	Lactate dehydrogenase	192 IU/L
Eosinophil	17.4%	Alkaline phosphatase	82 IU/L
Monocyte	5.1%	γ-glutamyl transpeptidase	24 IU/L
Hemoglobin	15.7 g/dl	Total protein	8.3 g/dL
Hematocrit	46.6%	Albumin	4.4 g/dL
Platelets	20.8/μl	Urea nitrogen	22 mg/dL
		Creatinine	0.95 mg/dL
		Sodium	139 mEq/L
Total IgE	529 IU/mL	Potassium	4.3 mEq/L
Antigen-specific IgE		Chloride	104 mEq/L
*Aspergillus*	≦0.34 UA/mL	Calcium	9.2 mg/dL
*Candida*	≦0.34 UA/mL	C-reactive protein	1.45 mg/dL
*Alternaria*	≦0.34 UA/mL	MPO-ANCA	(—) IU/mL

Abbreviation: IgE, immunoglobulin-E, RAST, radioallergosorbent test, MPO-ANCA, myeloperoxidase-anti-neutrophil cytoplasmic antibodies.

**FIGURE 2:**
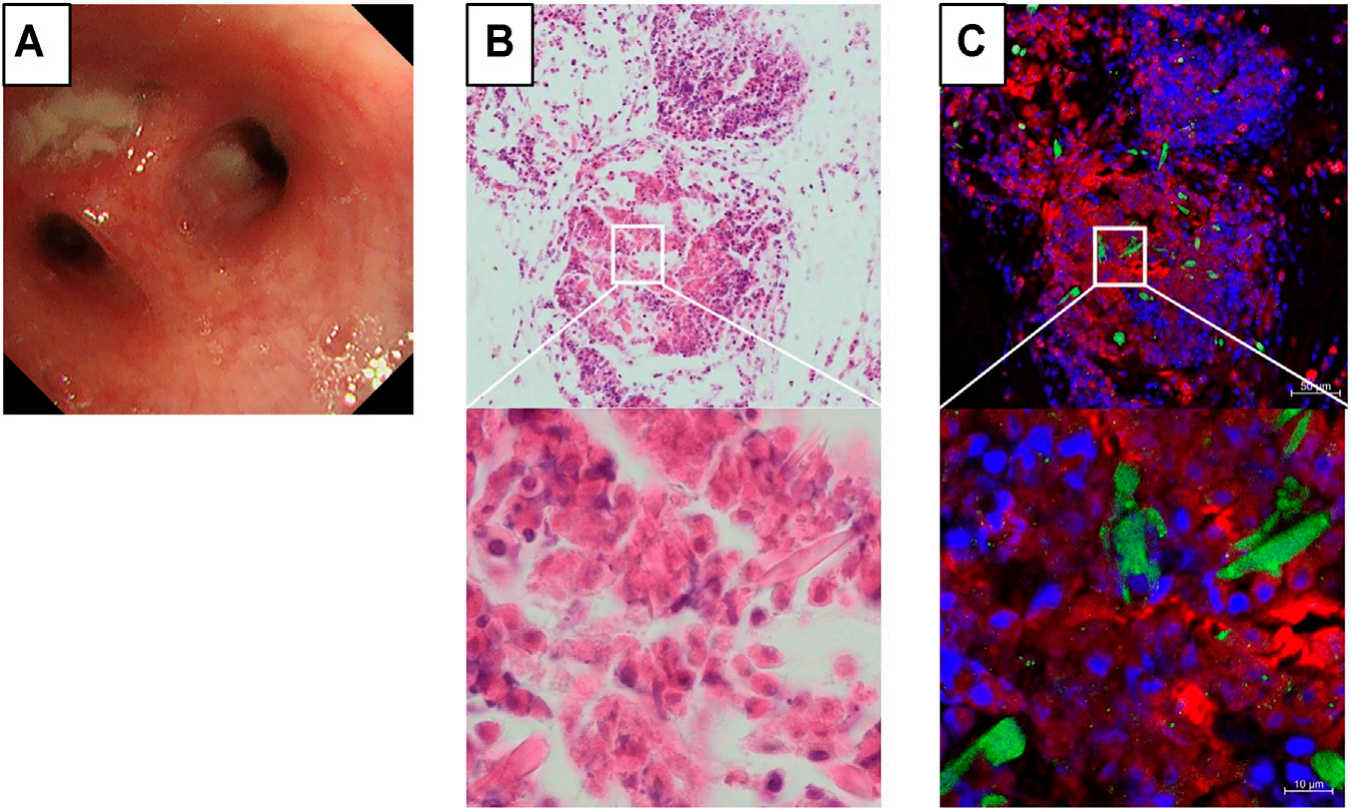
Immunostaining findings of the mucus plug on bronchoscopy. **(A)** An obstructive mucus plug is present in the bronchial lumen of B^1+2^c of the left upper lobe. **(B)** Hematoxylin and eosin staining of the mucus plug section showing a multilayered structure, with the accumulation of many chromatolytic eosinophils. **(C)** Formation of galectin-10-positive Charcot–Leyden crystals (green) and net-like DNA (blue) can be observed near intensive staining of the major basic protein (red). Identical section to B was immunostained.

Bronchoscopy showed obstruction of the subsegmental bronchus with mucus plugs (Figure 2A). The fraction of eosinophils in BAL fluid increased to 67%. The culture of the bronchoalveolar lavage specimen was positive for *Moraxella catarrhalis* and negative for *Mycobacterium tuberculosis* and fungi. Hematoxylin and eosin staining of the mucus plug section showed a multilayered structure with accumulation of many chromatolytic eosinophils (Figure 2B). On immunofluorescence staining of identical section (Fukuchi et al., 2021), there was deposition of major basic protein (MBP) over a wide area. Galectin-10-positive Charcot–Leyden crystals (CLCs) were formed near the MBP-deposited area with net-like DNA (Figure 2C). These immunostaining findings suggested that the deposition of granule proteins and the formation of CLCs proceeded because of the cytolytic process of eosinophils.

Based on these clinical findings, the possibility of eosinophilic granulomatosis with polyangiitis (EGPA), allergic bronchopulmonary mycosis (ABPM), and eosinophilic pneumonia was excluded. The diagnosis of eosinophilic bronchiolitis was established based on the reported diagnostic criteria (Poletti, 2013). Ceftriaxone (2 g/day) and oral prednisolone (30 mg/day) were initiated. After 4 days of treatment with these drugs, the respiratory symptoms with fever and hypoxia disappeared. His blood eosinophil count decreased to 165/μl after 14 days of treatment.

When the dose of oral prednisolone was tapered to 15 mg/day 3 months after disease onset, his respiratory symptoms worsened. HRCT showed bronchial wall thickening and centrilobular nodules, as seen during diagnosis (Figure 1B), suggesting recurrence of the disease. The dose of oral prednisolone was increased to 20 mg/day. Given the long-term adverse events due to systemic corticosteroids, benralizumab, an anti-IL-5Rα antibody, was added. After 14 days of its initiation, the blood eosinophil count decreased to 0/μl, and his respiratory symptoms improved. ACT score improved to 16. Radiological findings of bronchial wall thickening and centrilobular nodules also attenuated (Figure 1C). The dose of oral prednisolone was tapered to 15 mg/day without relapse.

## Discussion

According to previous reports (Poletti, 2013; Tang et al., 2015; Takeshita et al., 2020), systemic corticosteroids are required for the treatment of eosinophilic bronchiolitis owing to insufficient therapeutic effects of inhaled corticosteroids and bronchodilators. These reports indicate the necessity of maintenance therapy using systemic corticosteroids. Therefore, it is necessary to elucidate the pathogenesis of this refractory disease and establish a therapeutic strategy.

Eosinophil ETosis (EETosis) is a cytolytic process caused by cell death resulting from excessive activation of cells. During this process, intracellular chromatin structures and cytotoxic granule proteins, including MBP, are released (Asano et al., 2020). Recent studies have shown that galectin-10, an intracellular protein abundantly contained in eosinophils, crystallizes to form CLCs through EETosis. EETosis is observed in various eosinophilic disorders, including ABPM, eosinophilic chronic rhinosinusitis, EGPA, eosinophilic sialadenitis, and eosinophilic otitis media (Ohta et al., 2018; Fukuchi et al., 2021; Sano et al., 2021). Notably, it has not been reported in eosinophilic bronchiolitis, suggesting a pathogenic relationship between EETosis and the disease. However, the mechanism underlying EETosis development in eosinophilic bronchiolitis remains unclear.

The mucus plug in the subsegmental bronchus was infiltrated with cytolytic eosinophils with CLC formation in our case, indicating a pathogenic relationship between EETosis and mucus plug in eosinophilic bronchiolitis. Importantly, mucus plug and mucus hypersecretion are strongly associated with severe eosinophilic asthma (Dunican et al., 2018). Also, these manifestations are often accompanied with bronchiectasis with chronic airflow obstruction in severe asthma (Crimi et al., 2021). Thus, EETosis may be an optimal therapeutic target in refractory eosinophilic inflammation.

Benralizumab is a humanized monoclonal antibody targeting IL-5Rα that eliminates eosinophils by antibody-dependent cellular cytotoxicity through natural killer cells. In addition, a recent study revealed that the effective elimination of eosinophils by benralizumab was mediated by antibody-dependent cell phagocytosis activity during cell–cell interactions between macrophages and apoptotic eosinophils (Dagher et al., 2021). In the Phase III SIROCCO trial (NCT01928771) and CALIMA trial, benralizumab significantly reduced asthma exacerbations and improved lung function and respiratory symptoms in patients with uncontrolled severe eosinophilic asthma (Eugene et al., 2016; Mark et al., 2016). Of note, benralizumab produced a 95.8% reduction in airway eosinophils, whereas mepolizumab, a humanized monoclonal antibody targeting IL-5, reduced them by 55% (Flood-Page et al., 2003; Laviolette et al., 2013). In addition, benralizumab might be effective for the treatment of acute exacerbation of severe eosinophilic asthma without the use of systemic corticosteroids (Nolasco et al., 2020). These findings confirm that benralizumab is a good therapeutic option for the treatment of severe eosinophilic asthma.

In a previous case of ABPM, switching from mepolizumab to benralizumab resulted in rapid disappearance of mucus plugs (Tomomatsu et al., 2020). In addition, the therapeutic efficacy of benralizumab has been demonstrated in patients with EETosis-occurring eosinophilic annular erythema and chronic eosinophilic pneumonia (Masaki et al., 2021; Takano et al., 2021). Although a reported case of steroid-resistant eosinophilic bronchiolitis showed good therapeutic response to mepolizumab, benralizumab might be an additional option for the treatment of eosinophilic bronchiolitis.

The advantage of biologics is that they reduce the dose of systemic corticosteroids to be administered and decrease the risk of long-term adverse events (Bel et al., 2014; Nair et al., 2017). Importantly, long-term treatment with systemic corticosteroids in patients with asthma showed a hazard ratio of 1.34 for mortality compared with steroid-free treatment (Magnus et al., 2019). Previous reports and our case indicate the need for long-term systemic corticosteroid therapy in the management of eosinophilic bronchiolitis. Because the patient was a young adult in this case, the addition of biologics to the treatment regimen was strongly recommended for better outcomes throughout his life.

This is the first demonstration of EETosis in the mucus plugs of a patient with eosinophilic bronchiolitis who was successfully treated with benralizumab. However, this case report has several limitations. First, the role of inflammatory cells other than eosinophils has not been fully discussed. Second, this is a representative case to show the possibility of benralizumab as a therapeutic option in this disease. Thus, randomized, controlled, double-blinded clinical trials are needed to demonstrate the efficacy of benralizumab in clinical settings.

In conclusion, we report a case of eosinophilic bronchiolitis that was controlled with systemic corticosteroids and the additive benralizumab. Benralizumab may be effective for EETosis-occurring eosinophilic disorders with resistance to standard treatment and systemic corticosteroids. Further investigation is needed to establish an optimal therapeutic strategy using benralizumab in eosinophilic bronchiolitis.

## Data Availability

The original contributions presented in the study are included in the article/supplementary material, further inquiries can be directed to the corresponding author.
